# Subclinical Infection with Avian Influenza A H5N1 Virus in Cats

**DOI:** 10.3201/eid1302.060608

**Published:** 2007-02

**Authors:** Michael Leschnik, Joachim Weikel, Karin Möstl, Sandra Revilla-Fernández, Eveline Wodak, Zoltan Bagó, Elisabeth Vanek, Viviane Benetka, Michael Hess, Johann G. Thalhammer

**Affiliations:** * University of Veterinary Medicine, Vienna, Austria; † Austrian Agency for Health and Food Safety Institute for Veterinary Disease Control, Mödling, Austria; Subclinical Avian Influenza H5N1 Infection in Cats

**Keywords:** Cats, influenza A, virus subtype H5N1, natural infection, transmission, birds, susceptibility, research

## Abstract

Avian influenza A virus subtype H5N1 was transmitted to domestic cats by close contact with infected birds. Virus-specific nucleic acids were detected in pharyngeal swabs from 3 of 40 randomly sampled cats from a group of 194 animals (day 8 after contact with an infected swan). All cats were transferred to a quarantine station and monitored for clinical signs, virus shedding, and antibody production until day 50. Despite unfamiliar handling, social distress and the presence of other viral and nonviral pathogens that caused illness and poor health and compromised the immune systems, none of the cats developed clinical signs of influenza. There was no evidence of horizontal transmission to other cats because only 2 cats developed antibodies against H5N1 virus.

## Abstract 2

Infection without disease may occur under natural conditions after contact with infected birds.

Avian influenza has attracted worldwide attention because highly pathogenic avian influenza virus subtype H5N1 can cause fatal infections in humans (*1*) and other mammals (*2*). Domestic cats and wild cats in a zoo have reportedly shown severe clinical signs and they may die of natural or experimental infections (*3*–*7*). Ingestion of infected birds was assumed to be the route of transmission in cats. However, horizontal transmission by experimentally infected cats has been demonstrated (*3*) and was also assumed under natural conditions in tigers in Thailand (*8*). No data are available on nonlethal outcomes of H5N1 infection in cats and whether horizontal transmission between feline hosts occurs under natural conditions. Also unknown is whether domestic cats play a role in the epidemiology of avian influenza, which could be an undefined hazard for poultry and humans (*9*).

During the first weeks of 2006, moribund or dead birds infected with avian influenza H5N1 were found near water in Germany, Slovenia, and Austria. On February 14, 2006, a sick swan was found near the Mur River in Austria and transported to an animal shelter in Graz, Austria, where it died within 24 hours (day 1). PCR and egg culture identified avian influenza virus (H5N1) in the swan and in 13 of 38 other culled birds (swans, ducks, chickens) (day 4) housed with the swan at the same time. Only the swan developed clinical signs of disease. On day 4, the poultry area was disinfected after all 38 birds were removed.

In the same shelter were 194 cats; most had access to an outdoor enclosure near the poultry area and were separated from the birds by a wire-mesh fence. On several occasions, 1 or 2 unidentified cats were observed climbing the fence and entering the poultry area. Ingestion of birds by cats was not observed. Austrian authorities ordered random sampling of the cat population at the shelter because of spatial proximity of poultry and cats and the possible exposure of cats to infective debris of the birds. The bird area was left unoccupied while the cats were under observation. The purpose of this study was to monitor health status and possible transmission within a large cat population with proven natural exposure to H5N1 influenza virus.

## Materials and Methods

Pharyngeal swabs of 40 cats were sampled (*10*) on day 8 and tested for H5N1 virus by PCR; positive results were obtained for 3 cats (cats 1, 2, and 3). All positive results were confirmed at the OIE reference laboratory in Weybridge, United Kingdom. All PCRs for H5N1 were conducted at the Agency for Health and Food Safety in Mödling, Austria. Daily physical examination by veterinarians showed no signs of influenza in any cat on days 4–21. In a follow-up examination on day 15, 0 of 34 cats of the 40 cats previously tested (on day 8) were positive for H5N1 virus in pharyngeal swabs. In 3 cats that had died during this period, necropsy showed no evidence of infectious respiratory disease, and PCR results for influenza virus were negative.

On day 22 after the H5N1-infected swan was put in the animal shelter, 167 cats (5 kittens 4–6 months of age and 162 adults) were still available for further observations. Three cats had died and 24 other cats had been placed in private households. Before discharge from the shelter and within 1 week thereafter, all of these cats were examined and no abnormal health status was observed.

A total of 167 cats were transported in small groups in ≈50 containers for 12 h from the animal shelter to a quarantine area and housed in 2 separate groups from day 22 until day 50. Average floor space for each cat was ≈1.4m^2^. The larger group contained 139 cats (including cats 1 and 2); the smaller group contained 28 cats. Cat 3 was not available for further examination because it was healthy before leaving the shelter and, to our knowledge, did not die. The smaller group was always separated from the larger group and was kept indoors at the animal shelter in Graz. In the quarantine area, the 167 cats were housed in 2 closed rooms, without any activity restriction, and had free access to food and water. Routine physical examination, including auscultation of the chest, was done on days 22, 29, and 50 for all cats at the quarantine station. In case of an obvious health problem, clinical signs were monitored by daily physical examination and serologic testing. The litter pans of the cats and floors of the quarantine areas were cleaned every day and disinfected every other day.

On days 22 and 29, pharyngeal and rectal swabs were obtained and transported in phosphate-buffered saline containing antimicrobial drugs (*10*). Swabs were obtained with special care to avoid any contact with the environment and were transferred immediately into tubes containing transport media. Blood was obtained on days 22, 29, 36, and 50. To facilitate physical examinations and collection of samples, we gave mild general anesthesia (propofol and midazolam) to all cats on day 29 (Table).

**Table:** Time scale of sampling and course of influenza A virus H5N1 hemagglutinating antibody titer in cats 1, 2, and 4, Austria, 2006*

Day	Timeline events	No. pharyngeal swabs	No. rectal swabs	No. blood samples	H5N1 antibody titer
1	H5N1 virus–positive swan dies				
4	All poultry culled (38); 13 positive				
8	3 cats positive	40			
15	All cats negative	34			
22	Quarantine starts	160	160	14	Cat 1, 64 Cat 2, negative Cat 4, ND
29	Examination under anesthesia	All cats (164)	164	164	Cat 1, 128 Cat 2, negative Cat 4, 256
36				28	Cat 1, 128 Cat 2, negative Cat 4, ND
50	Cats 1 and 4 humanely killed for necropsy			All cats (155)	Cat 1, 256 Cat 4, 256

*ND, not done.

Pharyngeal and rectal swabs were examined for the matrix gene of influenza A virus by using a real-time reverse transcription–PCR (RT-PCR) according to the method of Spackman et al. (*11*). To screen for additional infections that might influence the health and immune status of the cats, we obtained 64 additional pharyngeal swabs on day 29 from cats with upper respiratory symptoms and tested them for nucleic acids specific for feline herpesvirus 1 (FHV-1)– and feline calicivirus (FCV). Real-time RT-PCR for FCV was conducted in a volume of 25 μL (22 μL reaction mixture and 3 μL template) in the Real-Time PCR system 7300 (Applied Biosystems, Foster City, CA, USA). The reaction mixture was prepared following the manufacturer’s instructions of a commercially available kit (SuperScript III Platinum One-Step Quantitative RT-PCR Kit, Invitrogen, Carlsbad, CA, USA). This mixture contained 10 pmol/L of each primer (forward primer: 5′-agtggcatgaccgccct-3′, reverse primer: 5′-cgttagcgcaggttgagca-3′), and 5 pmol/L of probe (5′-FAM-cactgtgatgtgttcgaagtttgagca-TAMRA-3′). The cycler scheme consisted of 2 pre-PCR steps of 50°C for 15 min and 95°C for 2 min, followed by 45 cycles of 95°C for 15 s and 60°C for 30 s. Cycle threshold values were calculated by using PCR 7300 software (Applied Biosystems). FHV-1 nucleic acid was detected by PCR as described by Reubel et al. (*12*) and Stiles et al. (*13*).

Plasma samples were tested for feline leukemia virus (FeLV) antigen and antibodies against influenza virus A H5N1, feline immunodeficiency virus (FIV), and feline coronavirus (FCoV). Antibodies to influenza virus were detected with a hemgglutination inhibition test according to the procedures of the World Organisation for Animal Health (*14*), FeLV antigen was detected by using an ELISA (ViraCHEK/FeLV, Synbiotics Corporation, San Diego, CA, USA), and antibodies to FIV were detected by using an immunomigration test (Witness FIV, Synbiotics Corporation). Three dilutions (1:10, 1:100, and 1:400) of each plasma sample were tested for antibodies to group 1 coronaviruses by a modified indirect immunofluorescence assay (*15*). Conjunctival, pharyngeal, and rectal swabs were cultured for pathologic bacterial infections (*16*).

Two cats that seroconverted for H5N1 virus (cats 1 and 4) were humanely killed on day 50. Necropsy was performed on these 2 cats and on 12 other cats that had died during the observation period; organ homogenates (lung, liver, brain, trachea, tonsils, stomach, spleen, and pancreas) were tested for influenza virus–specific nucleic acids for each cat.

## Results

H5N1 virus–positive cats (1 and 2) and H5N1 virus antibody–positive cats (1 and 4) did not show any signs of influenza virus–associated illness after the swan had been placed in the animal shelter (days 1–50). Upper respiratory symptoms (laryngitis, bronchitis, and tracheitis) were evident in 30 cats, bronchopneumonia in 40 cats, diarrhea in 7 cats, mucosal lesions in 37 cats, and traumatic wounds and lesions in 10 cats. However, for each cat with clinical symptoms that might have been associated with influenza infection, another specific etiologic reason for illness could be documented. Pathomorphologic examination showed no lesions associated with respiratory infection in cats 1 and 4 or in any other cat that had died before day 50. Influenza A virus–specific nucleic acids were not detected in any organ sample tested by PCR. Likewise, all pharyngeal and rectal swabs obtained at the quarantine station were negative for influenza A virus by PCR. Antibodies against influenza virus A H5N1 were detected in 2 cats (1 and 4, Table) with titers 256 on day 50 in both cats.

Cats 1, 2, and 4 had negative test results for FeLV and FIV, but all 3 cats had high antibody titers against FCoV. FCV was detected in the swab from cat 2, and a double infection with FCV and FHV-1 was detected in cat 4. Clinical, bacteriologic, and virologic tests identified infection with FeLV in 15 cats, FIV in 12 cats, and antibodies against FCoV in all but 1 cat. A total of 44 swabs showed positive results for FCV-specific nucleic acids, 4 for FHV-1; 13 samples showed a double infection with FCV and FHV-1. Some pathologic bacterial infections of the respiratory and digestive system were confirmed by swab cultures.

All veterinarians and staff members at the animal shelter and at the quarantine area were clinically monitored for any influenzalike symptoms. Because results of this monitoring were unremarkable and virus excretion by the cats was not detected, serologic tests were not conducted for these persons.

## Discussion

This is the first description of an asymptomatic infection with highly pathogenic H5N1 influenza virus in domestic cats. Although infection was detected in a group of cats by positive PCR results for pharyngeal swabs in 3 cats and seroconversion in 2 cats, there was no evidence for influenza-associated disease. This finding contrasts with reports documenting cats with rapidly and developing and fatal disease caused by influenza A virus subtype H5N1 (*3*–*5*,*7*). High fever, depression, severe pneumonia, pulmonary edema, nonsuppurative encephalitis, and sudden death were observed after natural (*5*) or experimental infections (*3*,*7*). Infection with influenza virus H5N1 was shown to cause severe lower respiratory tract disease as well as systemic disease that affected many organs outside the respiratory tract, which could explain the increased pathogenicity of this virus for other organ systems (*3*,*17*).

During the observation period, episodes of sickness including respiratory symptoms (mild dyspnea, conjunctival, and nasal discharge), oral mucosal lesions, and diarrhea were observed in cats in both groups in the animal shelter and in the quarantine station. A long (12 hours) and uncomfortable transport to the quarantine area, social distress caused by high population density, repeated restraint for examinations and sample collection, and multiple infectious agents may have caused such a high level of illness. Twelve cats died or were humanely killed while in a moribund state between days 22 and 50. All showed signs of disease other than infection with influenza virus A and died of feline infectious peritonitis, cardiomyopathy, enteritis, or nephropathy; none tested positive for H5N1 virus.

During the observation period from days 22 to 50, excretion of virus was not detected in the pharynx or feces. Positive results were observed only on day 8 for 3 of the randomly sampled swabs. Therefore, viral shedding is assumed to have lasted <2 weeks in cats 1 and 2. In 1 study, no information was reported on the duration of virus shedding because only severe illness with a lethal outcome was reported or the cats were killed 7 days after experimental infection (*3*). Because seroconversion was confirmed in only 2 animals, horizontal transmission within the group of 194 cats is unlikely. This conclusion is consistent with the finding that no virus shedding could be demonstrated after day 8, but it contrasts with the results of Rimmelzwaan et al. (*3*), who demonstrated horizontal transmission from experimentally infected cats to sentinel cats, and results of studies in mice and ferrets (*18*,*19*). After infection by the oral or intratracheal route, cats developed viremia; virus spread into different tissues and was excreted in feces and saliva (*7*). High viral load and differences in virus strains could result in different host reactions.

The reason for limited horizontal transmission in our study could be low-level virus shedding by the initially infected cats. Initial virus load, route of virus uptake, and the immune system of the cat may affect infection and disease. Otherwise, the lack of illness would be unusual because several cats in the study had immunodeficiencies caused by other infectious diseases (*20*,*21*).

An asymptomatic infection confirmed by seroconversion is assumed for cats 1 and 4. The situation for cat 2 is not as clear. It is unlikely that the positive PCR result in the swab sample from cat 2 is due to contamination and is a false-positive result. Conversely, infection could not be confirmed by seroconversion. It remains unclear whether ongoing infection could be stopped (possibly by interferons) or whether the cat did not produce sufficient amount of antibodies. Little information is available on immune responses after infection with influenza virus H5N1 in cats.

H5N1 virus can cross species barriers (*22*) and infect new hosts. Transmission from poultry to mammals, between cats (*3*,*5*,*7*,*8*), and between humans (*23*) indicates 2 routes of virus uptake under natural conditions. The first is orally by ingestion of raw poultry, and the second is transmission by contact with feces or saliva of infected animals. In our study, virus transmission from infected poultry to cats must have occurred from days 1 to 4. Uptake of H5N1 virus by ingestion of infected poultry can be ruled out. We observed only some cats entering the area where the birds were housed. Therefore, the most likely route of transmission for these cats is contagious fecal contamination of the hair and oral uptake during grooming. However, we cannot exclude aerosolization of the virus as a route of transmission.

Until recently, the avian flu situations in Asia and Europe appeared to differ. In Asia, large numbers of poultry have been infected and culled. Human and feline cases are mainly associated with close contact with infected poultry or ingestion of contaminated meat that was not sufficiently cooked. In Europe, mainly wild aquatic birds were infected, and only a few turkey farms were affected by H5N1 infection. Because direct contact with poultry is more limited in Europe than in Asian countries and the main source of food for cats in Europe is either commercial cat food or wild rodents and small birds, virus uptake during hunting and ingestion of poultry and aquatic birds is unlikely. Large aquatic birds are normally not a major source of food for cats, although infected birds may have caused the deaths of 3 cats found on the island of Ruegen, Germany (*4*).

We have shown that under natural conditions infection of cats with influenza virus H5N1 may occur after contact with infected birds or their excrement without inducing clinical disease. However, horizontal transmission between cats was not observed, although infected cats had been introduced into a large cat population that had other viral and bacterial infections and lived under stressful conditions. Avian flu infection in cats is rarely documented and there is no evidence to date that cats are responsible for transmitting the virus to humans. Although this study does not rule out H5N1 infection leading to disease and possible transmission to other mammals and birds by domestic cats under natural conditions, without ingestion of infected birds, cats do not represent a major risk in the epidemiology of H5N1 influenza. The risk posed by cats could change because the virus can rapidly undergo genetic mutation and reassortment, and efforts should be made to minimize contact of domestic cats with infected birds. To have better insights into whether cats represent a potential risk in the epidemiology of H5N1 influenza, more detailed knowledge is needed about the role of viral load, virus uptake, and immune mechanisms of the host on the outcome of infection with H5N1 influenza virus.
